# Decoding the Anti-*Trypanosoma cruzi* Action of HIV Peptidase Inhibitors Using Epimastigotes as a Model

**DOI:** 10.1371/journal.pone.0113957

**Published:** 2014-12-02

**Authors:** Leandro S. Sangenito, Rubem F. S. Menna-Barreto, Claudia M. d′Avila-Levy, André L. S. Santos, Marta H. Branquinha

**Affiliations:** 1 Laboratório de Investigação de Peptidases, Departamento de Microbiologia Geral, Instituto de Microbiologia Paulo de Góes (IMPG), Universidade Federal do Rio de Janeiro (UFRJ), Rio de Janeiro, Brazil; 2 Laboratório de Biologia Celular, Instituto Oswaldo Cruz (IOC), Fundação Oswaldo Cruz (FIOCRUZ), Rio de Janeiro, Brazil; 3 Laboratório de Biologia Molecular e Doenças Endêmicas, IOC, FIOCRUZ, Rio de Janeiro, Brazil; 4 Programa de Pós-Graduação em Bioquímica, Instituto de Química, UFRJ, Rio de Janeiro, Brazil; Federal University of São Paulo, Brazil

## Abstract

**Background:**

Aspartic peptidase inhibitors have shown antimicrobial action against distinct microorganisms. Due to an increase in the occurrence of Chagas' disease/AIDS co-infection, we decided to explore the effects of HIV aspartic peptidase inhibitors (HIV-PIs) on *Trypanosoma cruzi*, the etiologic agent of Chagas' disease.

**Methodology and Principal Findings:**

HIV-PIs presented an anti-proliferative action on epimastigotes of *T. cruzi* clone Dm28c, with IC_50_ values ranging from 0.6 to 14 µM. The most effective inhibitors, ritonavir, lopinavir and nelfinavir, also had an anti-proliferative effect against different phylogenetic *T. cruzi* strains. The HIV-PIs induced some morphological alterations in clone Dm28c epimastigotes, as reduced cell size and swollen of the cellular body. Transmission electron microscopy revealed that the flagellar membrane, mitochondrion and reservosomes are the main targets of HIV-PIs in *T. cruzi* epimastigotes. Curiously, an increase in the epimastigote-into-trypomastigote differentiation process of clone Dm28c was observed, with many of these parasites presenting morphological alterations including the detachment of flagellum from the cell body. The pre-treatment with the most effective HIV-PIs drastically reduced the interaction process between epimastigotes and the invertebrate vector *Rhodnius prolixus.* It was also noted that HIV-PIs induced an increase in the expression of gp63-like and calpain-related molecules, and decreased the cruzipain expression in epimastigotes as judged by flow cytometry and immunoblotting assays. The hydrolysis of a cathepsin D fluorogenic substrate was inhibited by all HIV-PIs in a dose-dependent manner, showing that the aspartic peptidase could be a possible target to these drugs. Additionally, we verified that ritonavir, lopinavir and nelfinavir reduced drastically the viability of clone Dm28c trypomastigotes, causing many morphological damages.

**Conclusions and Significance:**

The results contribute to understand the possible role of aspartic peptidases in *T. cruzi* physiology, adding new *in vitro* insights into the possibility of exploiting the use of HIV-PIs in the clinically relevant forms of the parasite.

## Introduction

Chagas' disease is known as a neglected tropical disease, and as such does not have the proper attention and funding from institutions and pharmaceutical industries [1]. The severe chronic phase of this disease includes myocarditis and complications in the digestive tract [2]. *Trypanosoma cruzi*, the etiologic agent of Chagas' disease, infects 8 million people in Latin America and has a wide distribution in these countries [3]. However, the recent influx of immigrants from endemic countries have turned Chagas' disease into a major health problem in the United States of America, Canada and in many parts of Europe, where an increasing number of infected individuals has been identified. Nowadays, in United States of America, it is estimated that 300,000 individuals are infected with *T. cruzi*. Among the non-endemic countries, Spain has the second highest number of infected habitants, mostly originating from Ecuador, Argentina, Bolivia and Peru [4], [5].

The changes in the epidemiology of Chagas' disease facilitate co-infection with human immunodeficiency virus (HIV) in areas with high viral prevalence, making diagnosis and prognosis even more difficult [3]. *T. cruzi*/HIV co-infection leads to reactivation of the parasitic infection, with exacerbation of clinical signs and unusual chronic phase manifestations, when the patient is subjected to an immunosuppression state [3], [6]. Although the involvement of the central nervous system is never observed in the chronic phase of Chagas' disease, it does occur in immunocompromised individuals, as a result of the reactivation of infection with *T. cruzi*, purchased years before [3], [7].

Few drugs are useful in clinical trials against *T. cruzi*, including benznidazole (a nitroimidazol) and nifurtimox (a 5-nitrofuran) [2]. However, the available chemotherapy for treating chagasic patients is unsatisfactory due to the numerous and severe side effects. Allied to this, parasite drug resistance emerges as a relevant fact to be considered. Therefore, the identification of biochemical/metabolic differences between parasites and their hosts undoubtedly provides a reasonable alternative for the development of new chemotherapeutic agents [8].

The characterization of peptidases is of interest to understand their characteristics and also to assess their roles in parasitic infections, exploring them as new chemotherapeutic targets [9]. In this context, aspartic peptidases have been identified in different classes of infectious agents, participating in various physiological and pathological events [10], [11]. However, the only aspartic peptidase inhibitors approved for chemotherapy are the ones used in anti-HIV therapy [10]. Our research group has focused in understanding the role of aspartic peptidases in the biology and life cycle of *T. cruzi*. We initially explored the effects of pepstatin A, a classical aspartic peptidase inhibitor, on the parasite development. Pepstatin A arrested the proliferation of epimastigotes, in both dose- and time-dependent manner. The treatment of parasite with pepstatin A resulted in significant morphological alterations, as detected by light microscopy analysis [12]. Nevertheless, pepstatin-like drugs are not used clinically because of their metabolism in the liver and rapid clearance from blood [13]. *T. cruzi* genome contains three aspartic peptidase genes, of which none has been further characterized [11], although aspartic peptidase activity was previously detected in epimastigote extracts [14].

In the present study, we have tested the effects of different HIV aspartic peptidase inhibitors (HIV PIs), which are used in the clinical arena, on the modulation of crucial biological events of *T. cruzi* epimastigotes, such as: proliferation, morphology, differentiation, aspartic peptidase activity, interaction with explanted guts from the insect vector *Rhodinus prolixus* and the expression of surface peptidases other than aspartic peptidases, including cruzipain (the major cysteine peptidase), gp63 (a zinc-metallopeptidase) and calpain (a calcium-dependent cysteine-type peptidase). In addition, we verified the effects of HIV PIs on the trypomastigote forms of the parasite.

## Methods

### Chemicals

The HIV PIs (amprenavir, indinavir, lopinavir, nelfinavir, ritonavir and saquinavir) were obtained through the National Institutes of Health (NIH) AIDS Research and Reference Reagent Program, Division of AIDS, NIAID. All the HIV PIs were dissolved in dimethylsulfoxide (DMSO) to obtain a final concentration of 8 mM and stored at −20°C until use. DMSO, cathepsin D substrate (7-methoxycoumarin-4-acetyl-Gly-Lys-Pro-Ile-Leu-Phe-Phe-Arg-Leu-Lys(DNP)-D-Arg-amide), ethylene diamine tetraacetic acid (EDTA), *trans*-epoxy succinyl L-leucylamido-(4-guanidino) butane (E-64), bovine serum albumin (BSA), 3-[(3-cholamidopropyl)-dimethylammonio]-1-propanesulfonate (CHAPS), fluorescein isothiocyanate (FITC)-labeled goat anti-rabbit Immunoglobulin G (IgG), anti-α-tubulin monoclonal antibody and Dulbecco's modified Eagle's medium (DMEM) were purchased from Sigma-Aldrich Chemical Co. (St Louis, USA). Fetal bovine serum (FBS) was obtained from Gibco Life Technology (New York, USA). Media constituents and buffer components were purchased from Amersham Life Science (Little Chalfont, UK). All other reagents were analytical grade.

### Parasites and cultivation

Epimastigote forms from different strains of *T. cruzi* (**Table 1**) were grown in Warren medium supplemented with 10% heat-inactivated FBS at 28°C for 4 days to reach late-log/stationary phase of growth [16].

**Table 1:** *Trypanosoma cruzi* strains used in this study, their major characteristics and HIV PIs IC_50_ values.

Isolates	Host origin	Geographicalorigin	Mini-exon typing	Typing groups a	IC_50_ values/72 h (µM)		
					Ritonavir	Lopinavir	Nelfinavir
Dm28c	*Didelphis marsupialis*	Venezuela	*T. cruzi* I	TcI	0.6	2.1	7.1
CL Brener	*Triatoma infestans*	Rio Grande do Sul, Brazil	*T. cruzi* II	TcVI	9.0	5.1	25.3
Y	*Homo sapiens*	São Paulo, Brazil	*T. cruzi* II	TcII	6.4	3.8	7.3
INPA 4167	*Rhodnius brethesi*	Brazilian Amazon region	Z3B	TcIV	7.4	4.3	6.9

a According to the new nomenclature [15].

### Effects of HIV PIs on the growth rate and cell morphology

The effects of six distinct HIV PIs (amprenavir, indinavir, lopinavir, nelfinavir, ritonavir and saquinavir) on *T. cruzi* clone Dm28c epimastigote forms were assessed by a method similar to that previously described elsewhere [12]. Briefly, epimastigotes were counted using a Neubauer chamber and resuspended in fresh medium to a final concentration of 5×10^6^ viable epimastigotes per milliliter. The viability was assessed by mobility and lack of Trypan blue staining. Each HIV PI was added to the culture at final concentrations ranging from 0.5 to 30 µM. After incubation for 24 to 96 h at 28°C the number of motile epimastigotes was quantified. The 50% inhibitory concentration (IC_50_) was determined after 72 h by linear regression analysis using Origin Pro 7.5 software. Light microscopy evaluation was performed in order to detect some possible alterations on parasite morphology after the treatment with HIV PIs [16]. In this context, the parasites were also stained with Giemsa and then observed in a Zeiss microscope (Axioplan, Oberkochen, Germany). By flow cytometry, each experimental population was then mapped by using a two-parameter histogram of forward-angle light scatter (FSC) versus side scatter (SSC), in order to measure two morphological parameters: cell size and granularity, respectively. Additionally, three other *T. cruzi* strains (Y, CL Brener and INPA 4167), belonging to distinct phylogenetic lineages (**Table 1**), were submitted to the treatment with the most efficacious HIV PIs for clone Dm28c and the IC_50_/72 h was also determined.

### Effects of HIV PIs on the parasite ultrastructure

Epimastigote forms from clone Dm28c (5×10^6^ cells/ml) were treated with HIV PIs at IC_50_ values for 72 h in Warren medium at 28°C. Afterwards, the parasites were fixed with 2.5% glutaraldehyde in 0.1 M sodium cacodylate buffer (pH 7.2) at room temperature for 40 min at 25°C and post-fixed with a solution of 1% OsO_4_, 0.8% potassium ferricyanide and 2.5 mM CaCl_2_ in the same buffer for 20 min at 25°C. Cells were dehydrated in an ascending acetone series and embedded in PolyBed 812 resin. Ultrathin sections were stained with uranyl acetate and lead citrate and examined in Jeol JEM1011 transmission electron microscope (Tokyo, Japan) at Plataforma de Microscopia Eletrônica, IOC, FIOCRUZ [17].

### Effects of HIV PIs on cellular differentiation

In order to quantify the effects of HIV PIs on this process, clone Dm28c epimastigote forms were treated with HIV PIs at 1 and 10 µM for 72 h and 96 h. The number of epimastigotes, differentiating forms and morphological forms typical of trypomastigotes was evaluated after Giemsa staining. The distinction between epimastigotes and trypomastigotes was performed by the analysis of the position of emergence of the flagellum in the parasite cell body and the position of the nucleus in relation to the kinetoplast and flagellar pocket [18]. In epimastigotes, the kinetoplast and the flagellar pocket are located anterior and near to the central nucleus, and a free flagellum emerges from the flagellar pocket. In trypomastigotes, the kinetoplast and the flagellar pocket display a posterior location and far from the central nucleus; the flagellum emerges from the flagellar pocket but stay adhered along the length of the cell body, becoming free only in the anterior region. The criterion to distinguish the differentiating forms was the relative positions of the nucleus, kinetoplast and flagellar pocket as not typical of either trypomastigotes or epimastigotes. At least 200 parasites were examined in each preparation in a Zeiss microscope (Axioplan).

An additional analysis was performed by flow cytometry in order to detect the glycoprotein gp82, a surface molecule that is unique and abundant in metacyclic trypomastigotes [19]. Briefly, parasites treated or not with HIV PIs (10 µM) for 96 h were fixed in 0.4% paraformaldehyde in PBS (pH 7.2) for 30 min at 4°C, followed by extensive washing in the same buffer. After this step, the parasites were incubated at room temperature for 1 h with a 1∶100 dilution of the anti-gp82 monoclonal antibody (kindly provided by Dr. Nobuko Yoshida – Department of Microbiology, Immunobiology and Parasitology, Universidade Federal de São Paulo, Brazil). Parasites were then incubated for an additional hour with a 1∶200 dilution of FITC-labeled goat anti-rabbit IgG. The cells were then washed three times in PBS and analyzed in a flow cytometer (FACS Calibur, BD Biosciences, USA) equipped with a 15-mW argon laser emitting at 488 nm. Non-treated cells and those treated with the secondary antibody alone were run in parallel as controls. The mapped population (10,000 events) was then analyzed for log green fluorescence by using a single parameter histogram [16].

### Aspartic peptidase measurement

The enzymatic activity over cathepsin D substrate was determined using *T. cruzi* epimastigote (clone Dm28c) extract, which was obtained by repeated freeze-thawing cycles of 10^8^ viable cells in a buffer containing 0.2 M sodium phosphate, 0.1 M citric acid, 1 mM EDTA, 1% CHAPS, 10 µM E-64, pH 4.0. Then, the cellular extract was incubated for 40 min at 4°C, centrifuged at 10,000×*g* for 30 min at 4°C, and the supernatant immediately used to determine the protein content and the proteolytic activity. The protein concentration was determined by the method described by Lowry and co-workers [20], using BSA as standard. The cleavage of cathepsin D substrate was monitored continuously in a spectrofluorometer (SpectraMax Gemini XPS, Molecular Devices, CA, USA) using an excitation wavelength of 328 nm and an emission wavelength of 393 nm. A 200 µM stock solution of the fluorogenic substrate was prepared in DMSO. The reaction was started by the addition of the substrate (2 µM) to the parasite extract (40 µg protein) in a total volume of 60 µl of a buffer containing 0.2 M sodium phosphate, 0.1 M citric acid, 1 mM EDTA, 10 µM E-64, pH 4.0, in the presence or the absence of each HIV PI at 10, 50 or 100 µM. The reaction mixture was incubated at 37°C for 2 h. The assays were controlled for self-liberation of the fluorophore over the same time interval [12].

### Effects of HIV PIs on the expression of peptidases

In this set of experiments, we evaluated the effects of HIV PIs on the expression of peptidases other than the aspartic-type in *T. cruzi*. Epimastigotes (5×10^6^ cells) of clone Dm28c were incubated with each HIV PI (IC_50_ value) for 24 h at 28°C. Thereafter, cells were processed for flow cytometry analysis, as mentioned before, in order to detect the well-known *T. cruzi* peptidases: cruzipain, gp63 and calpain-like molecules. The parasites were incubated at room temperature for 1 h with a 1∶250 dilution of the following rabbit polyclonal antibodies: anti-cruzipain, raised against a mixture of isoforms of natural cruzipain from the Tul 2 strain of *T. cruzi* (kindly provided by Dr Juan Jose Cazzulo – Instituto de Investigaciones Biotecnologicas, Universidad Nacional de General San Martin, Buenos Aires, Argentina); anti-gp63, raised against the recombinant gp63 molecule from *Leishmania mexicana* (kindly provided by Dr Peter Overath – Max-Planck-Institut für Biologie, Abteilung Membran Biochemie, Germany) and anti-Dm-calpain, raised against native calpain from *Drosophila melanogaster* (kindly donated by Dr Yasufumi Emori – Department of Biophysics and Biochemistry, Faculty of Sciences, University of Tokyo, Japan). Parasites were then incubated for an additional hour with the secondary antibody and analyzed in a flow cytometer [16].

The Western blotting analysis was performed as previously described by Sangenito and co-workers [16]. Epimastigotes (10^8^ cells) were collected and resuspended in 100 µl of PBS and lysed by the addition of 1% SDS. The protein concentration was determined by the method described by Lowry and co-workers [20]. Immunoblot analysis was performed with total cellular extracts equivalent to 100 µg of protein. The primary antibodies mentioned previously were used at a dilution of 1∶500 and the secondary antibody used was peroxidase-conjugated goat anti-rabbit IgG at a dilution of 1∶25,000 followed by chemiluminescence immunodetection after reaction with ECL reagents [16]. An anti-α-tubulin monoclonal antibody at 1∶500 dilution was also used as a control for sample loading in the immunoblot. The relative molecular mass of the reactive polypeptides was calculated by comparison with the mobility of SDS–PAGE standards and the densitometric analysis was performed using the ImageJ program.

### Effect of HIV PIs on the interaction of epimastigotes with *Rhodnius prolixus*

Specimens of *R. prolixus* used in this study were obtained from the insectary of the Laboratório Nacional e Internacional de Referência em Taxonomia de Triatomíneos, Instituto Oswaldo Cruz, FIOCRUZ, Rio de Janeiro, Brasil. The insects were kept at 28°C with 70% relative humidity and fed on rabbit blood feeding apparatus [21]. Fifth stage nymphs were collected randomly after the changes and fasted for 20 to 30 days. After this period, blood nymphs were fed in the same system and used between 10 and 14 days after feeding. The basic material for our studies was the midgut of nymphs of *R. prolixus*. Briefly, the insect gut was separated from the stomach and the rectum, and then stretched longitudinally opened with subsequent washing in PBS to remove feces. The guts were placed in 1.5-ml microcentrifuge tubes in PBS (final volume of 50 µl).

The epimastigote forms (clone Dm28c) were pre-treated or not with different concentrations of HIV PIs during 1 or 24 h, and in all cases cell viability was preserved. After the incubation periods, parasites (10^8^ cells) were washed 3 times in PBS and submitted to the interaction with the *R. prolixus* gut at room temperature for 1 h (final volume of 100 µl). The guts were extensively washed to remove non-adhered parasites, and then macerated to release the adhered forms [22]. The evaluation of the parasitic load was performed by counting in a Neubauer chamber, where the parasite viability was assessed by motility and exclusion of Trypan blue vital dye. The results are shown as the mean ± standard error (SE) of three independent experiments.

### Effects of HIV PIs on trypomastigote forms

Culture trypomastigote forms of clone Dm28c were obtained from infected LLC-MK_2_ cells after 5 days of incubation in DMEM supplemented with 2% heat-inactivated FBS at 37°C. The effects of the HIV PIs ritonavir, lopinavir and nelfinavir on *T. cruzi* trypomastigotes were assessed by incubation in DMEM with 2% heat-inactivated FBS. Briefly, trypomastigotes were counted using a Neubauer chamber and resuspended in fresh medium to a final concentration of 5×10^6^ viable cells per milliliter. The viability was assessed by motility and lack of Trypan blue staining. Each HIV PI was added to the culture at final concentrations ranging from 1 to 50 µM. After incubation for 24 h at 37°C, the number of motile parasites was quantified. The 50% lethal dose (LD_50_) was determined after 24 h by linear regression analysis using Origin Pro 7.5 software. Light microscopy evaluation was performed in order to detect some possible alterations on parasite morphology after the treatment with HIV PIs. In this context, the parasites were also stained with Giemsa and then observed in a Zeiss microscope (Axioplan, Oberkochen, Germany).

### Statistical analysis

All experiments were performed in triplicate, in three independent experimental sets. The data were analyzed statistically by means of Student's *t* test using EPI-INFO 6.04 (Database and Statistics Program for Public Health) computer software. *P* values of 0.05 or less were considered statistically significant.

## Results

### Effects of HIV PIs on the growth rate

**Figure 1:**
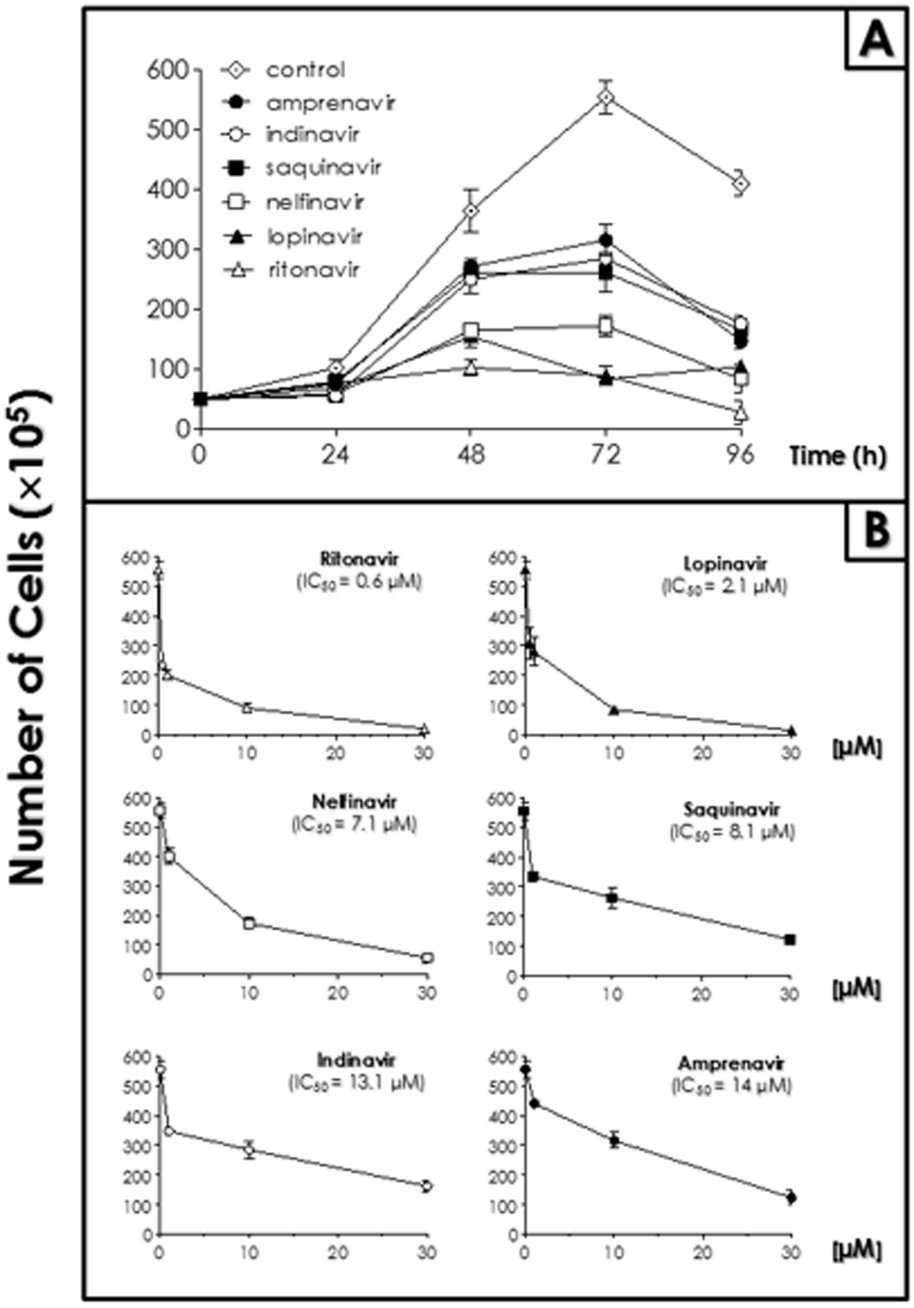
Effect of HIV PIs on the growth rate of *T. cruzi* clone Dm28c epimastigote forms. (A) The growth pattern was followed in the absence (control) and the presence of ritonavir, lopinavir, nelfinavir, saquinavir, indinavir and amprenavir at 10 µM for 96 h. (B) Ritonavir and lopinavir were assayed in concentrations ranging from 0.5 to 30 µM for 72 h, as well as nelfinavir, saquinavir, indinavir and amprenavir were tested in concentrations ranging from 1 to 30 µM for the same period. Viable cells were counted daily by Trypan blue exclusion and motility in a Neubauer chamber. Data shown are the mean standard error (S.E.) of three independent experiments performed in triplicate. The IC_50_ values were calculated after 72 h and are indicated for each drug. All HIV PIs inhibited parasite growth significantly after 48–96 h in relation to the control (*P*<0.05).

HIV PIs were effective in inhibiting the proliferation of epimastigotes of *T. cruzi* clone Dm28c in different extents, depending on both the compound and the concentration used. In the first case, all compounds were tested at 10 µM and all of them were able to inhibit the parasite growth for 96 h, although at different rates (**Fig. 1A**). After that, each inhibitor was tested in different and appropriated concentrations. The HIV PIs showed a typical dose-dependent inhibition after 72 h and the IC_50_/72 h values ranged from 0.6 µM (ritonavir) to 14 µM (amprenavir) (**Fig. 1B**). DMSO, the solvent used to dissolve the HIV PIs, had no effect on the parasite multiplication when added in the volume corresponding to the highest concentration of the HIV PIs (data not shown).

To determine whether HIV PIs would be effective against strains belonging to different *T. cruzi* lineages, the epimastigote forms of Y (TCII), INPA 4167 (TCIV) and CL Brener (TcVI) strains were submitted to the treatment with the three most efficacious inhibitors for clone Dm28c, i.e., ritonavir, lopinavir and nelfinavir. All the strains were sensitive to the inhibitors tested (**Table 1**). CL Brener strain was the most resistant strain, for which IC_50_ value was 3.5 times higher for nelfinavir in comparison to the IC_50_ value displayed for clone Dm28c. Similar IC_50_ values were found for Y and INPA 4167 strains (**Table 1**).

### Effects of HIV PIs on the cell morphology

**Figure 2:**
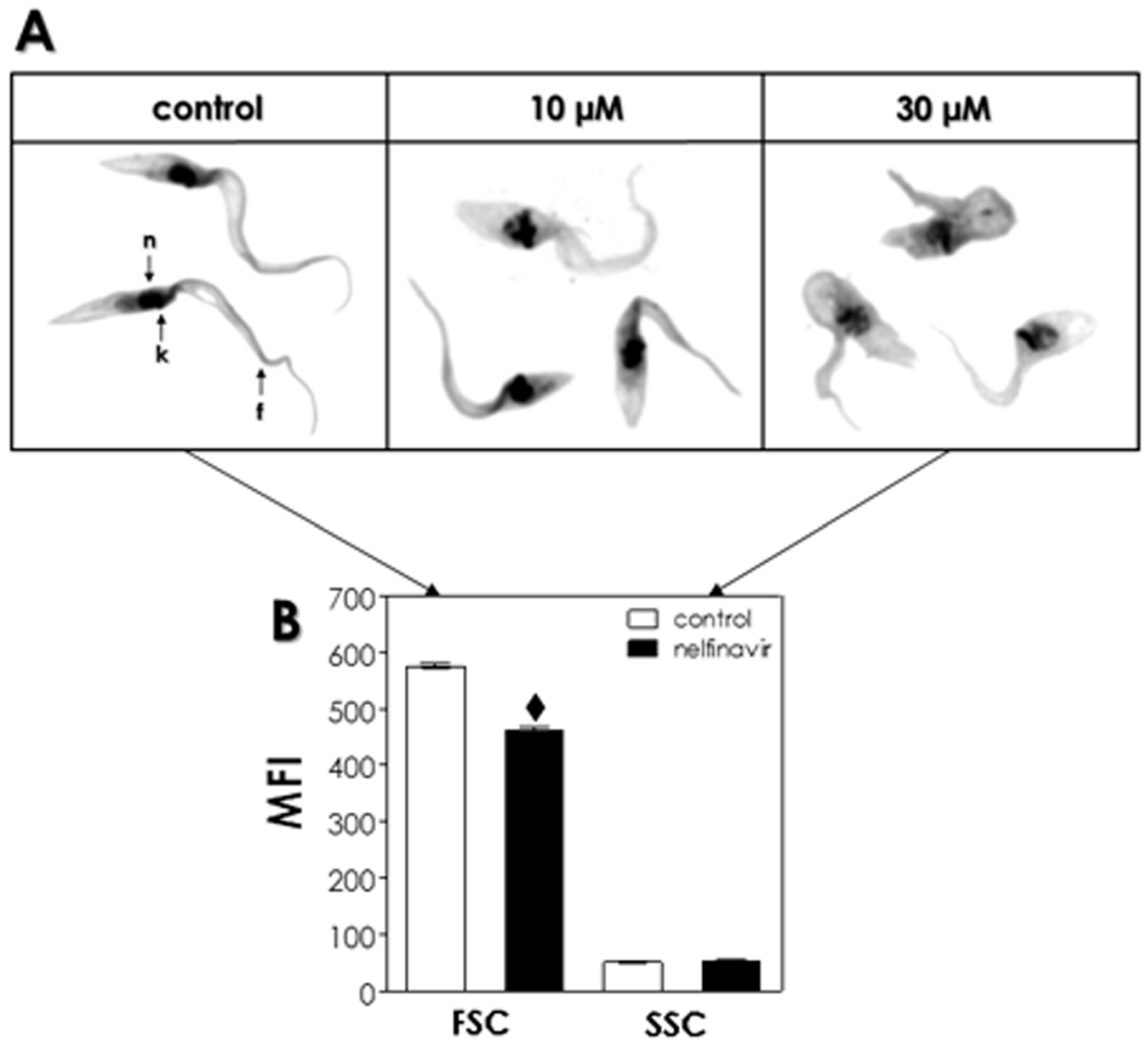
Effects of HIV PIs on morphology of *T. cruzi* clone Dm28c epimastigotes. (A) Giemsa-stained smears of *T. cruzi* epimastigote cells were incubated in the absence (control) or in the presence of nelfinavir at 10 and 30 µM for 72 h. Epimastigote forms present a kinetoplast (**k**) in the anterior end of the parasite, a central nucleus (**n**), an elongated cell body and a flagellum (**f**) attached to the parasite cell body. The microscopy images are representative of the treatment of epimastigotes with the six HIV PIs used in this work in concentrations higher than IC_50_ value. (B) Untreated parasites and those treated with nelfinavir (30 µM) for 72 h were subsequently analyzed by flow cytometry in order to measure two cellular parameters (size and granularity). Forward scatter (FSC) measurement is related to cell size and side scatter (SSC) measurement is related to the internal granularity and/or complexity of a cell that were expressed as mean of fluorescence intensity (MFI). The symbol (♦) denotes statistic difference to control (*P*<0.05).

Light microscopy analysis of clone Dm28c treated with the six HIV PIs at higher concentrations than the IC_50_ values revealed some morphological alterations in comparison to the typical epimastigote appearance: parasites became round in shape, with reduced cell size and swollen of the cellular body. In a representative set of these results, the treatment of parasite cells with nelfinavir (IC_50_ value 7.1) at 10 and 30 µM was shown (**Fig. 2A**). Corroborating some of these morphological alterations, HIV PIs-treated epimastigotes presented a significant reduction (around 20%) on the parasite size when compared to the untreated cells, as revealed by flow cytometry measurements. However, no alteration was observed on the granularity parameter (**Fig. 2B**).

### Effects of HIV PIs on the ultrastructure

**Figure 3:**
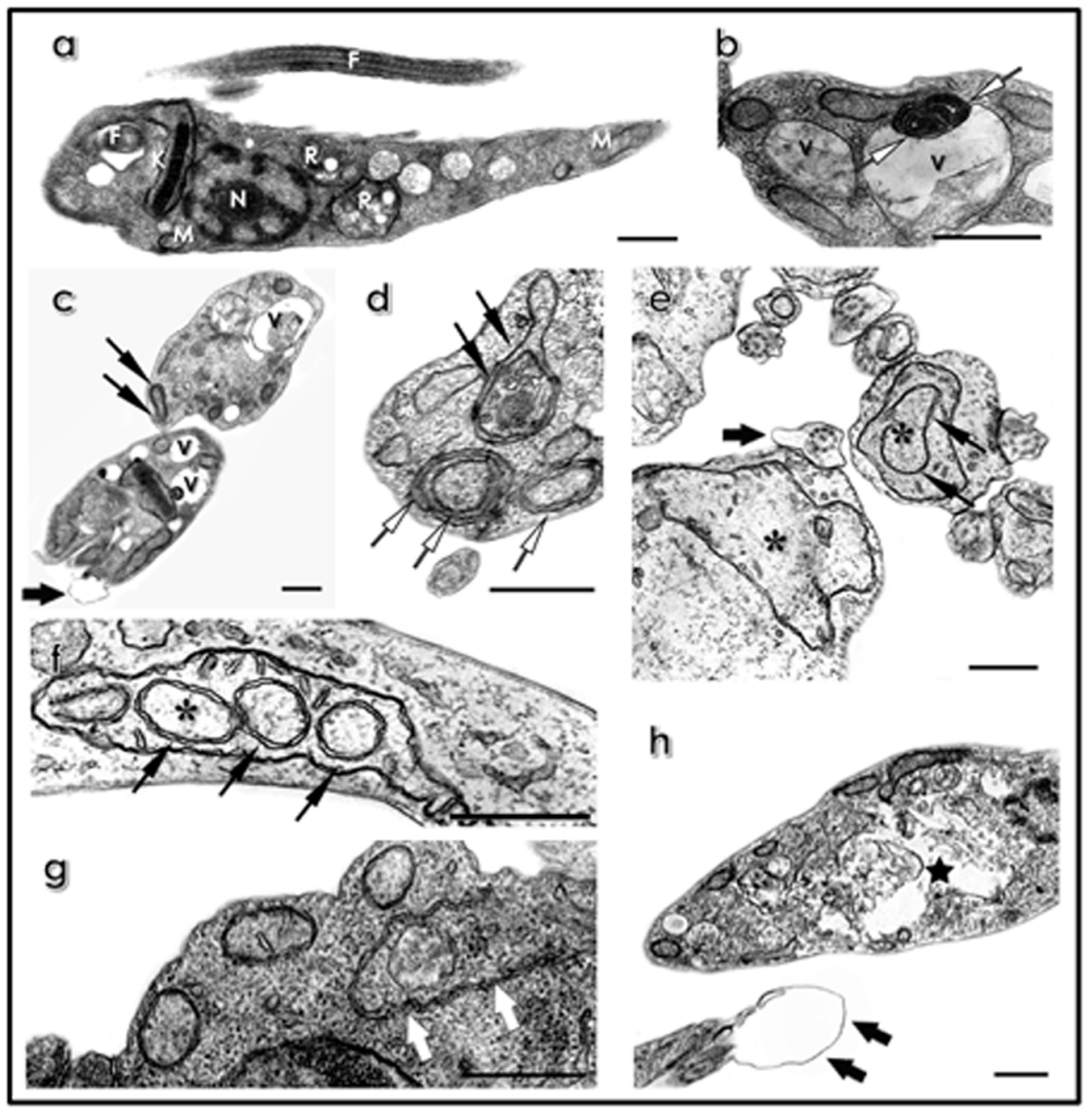
Ultrastructural effects of HIV PIs on *T. cruzi* clone Dm28c epimastigote forms. (**a**) Untreated parasite showing normal elongated morphology with typical nucleus (**N**), mitochondrion (**M**), flagellum (**F**), reservosomes (**R**) and kinetoplast (**K**). Parasites were treated with nelfinavir (**b**), indinavir (**c** and **d**), saquinavir (**e, f** and **h**) and amprenavir (**g**) at IC_50_ values for 72 h. **Black thick arrows**, blebbing in the flagellar membrane; **white arrows**, concentric membranar structures in the cytosol; **black arrows**, concentric membranar structures inside the mitochondrion; **white thick arrows**, endoplasmic reticulum profiles surrounding organelles and cytosolic structures; **asterisks**, mitochondrial swelling; **black star**, disruption of reservosomes; **V**, vacuolization with the presence of degraded material inside. Bars = 500 nm.

The treatment of clone Dm28c epimastigotes with all the six HIV PIs at IC_50_ values for 72 h led to strong and frequent formation of blebbing in the flagellar membrane (**Figs. 3c, e, h** **, black thick arrows**). These inhibitors also induced other cellular alterations, such as the presence of concentric membranar structures in the cytosol (**Figs. 3b, d** **, white arrows**) or inside the mitochondrion (**Figs. 3c, d, e, f** **, black arrows**), and an intense vacuolization with the presence of degraded material inside (**Figs. 3b, c, V**). For simplicity, these phenomena were represented only for nelfinavir (**Fig. 3b**), indinavir (**Fig. 3c, d**) and saquinavir (**Fig. 3e, f, h**). Particularly, the appearance of endoplasmic reticulum profiles surrounding organelles and cytosolic structures was only observed after the treatment with amprenavir (**Fig. 3g** **, white thick arrows**). Saquinavir was the unique inhibitor capable in promoting the mitochondrial swelling (**Figs. 3e, f** **, asterisks**) and the disruption of reservosomes (**Fig. 3h** **, black star**).

### Effects of HIV PIs on differentiation process

**Figure 4:**
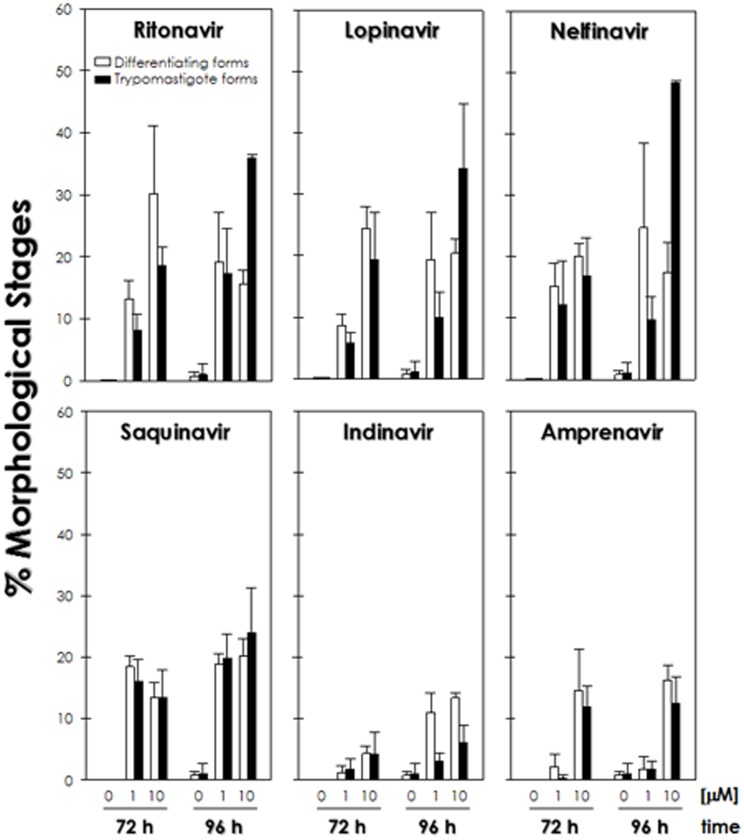
Effect of HIV PIs on the *T. cruzi* clone Dm28c epimastigote-into-trypomastigote differentiation. Epimastigotes were incubated for 72 h and 96 h in the absence or in the presence of HIV PIs at 1 and 10 µM. After this period, Giemsa-stained smears were prepared and the percentage of each morphological stage was calculated after counting at least 200 parasites for each system. The graphics show the percentage of differentiating forms (which was considered the intermediate morphological stage between typical epimastigotes and typical trypomastigotes) and trypomastigote cells. Data shown are the mean standard error (S.E.) of three independent experiments performed in triplicate. All values are statistically different from control except for: indinavir treatment for 72 h and after 96 h with 1 µM for trypomastigote forms; amprenavir treatment with 1 µM over 72 and 96 h (*P*<0.05).

**Figure 5:**
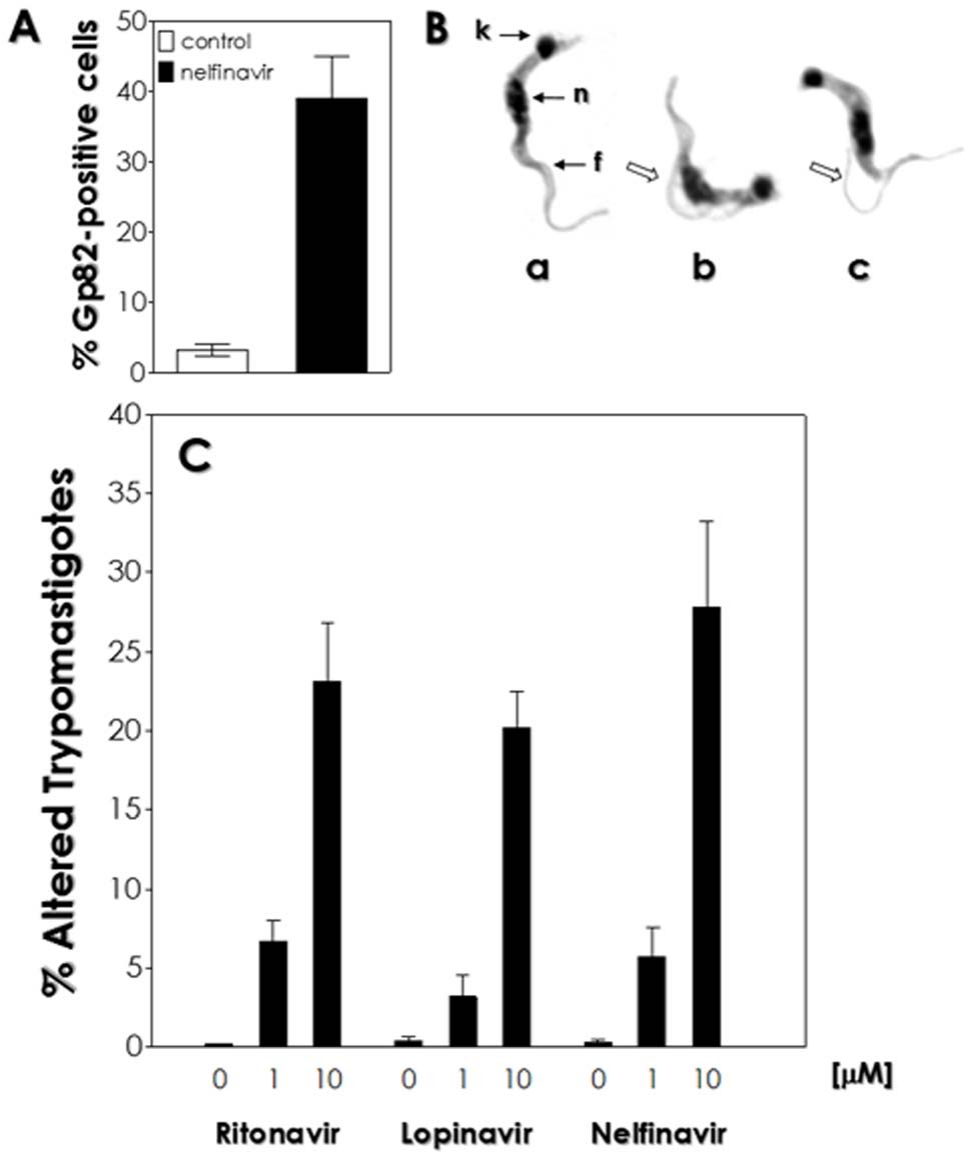
Detection of altered trypomastigote forms after cultivation of *T. cruzi* clone Dm28c epimastigotes in the presence of the HIV PIs ritonavir, lopinavir and nelfinavir. (A) Flow cytometric analysis of anti-gp82 antibody binding to *T. cruzi* trypomastigote-like forms after treatment with nelfinavir at 10 µM for 96 h. Data are expressed as the percentage of fluorescent cells. (B) Giemsa-stained smears were prepared, and the typical trypomastigote form can be seen in (a). Note that the presence of the HIV PIs induced the detachment of flagellum from the cell body (arrows in b and c). (k), kinetoplast; (f), flagellum; (n), nucleus. The images are representative of the three HIV PIs used with nelfinavir as a model. (C) The percentage of altered trypomastigote forms was calculated after counting at least 200 trypomastigotes for each system. Data shown are the mean standard error (S.E.) of three independent experiments performed in triplicate. All values are statistically different from control (*P*<0.05).

Curiously, HIV PIs were able to induce the differentiation process in Dm28c clone principally after 72 h and 96 h, in which epimastigotes transformed into intermediate and morphological forms typical of trypomastigotes (**Fig. 4**). Ritonavir, lopinavir, nelfinavir and saquinavir were the best inducers of the differentiation process, generating around 43 to 65% of differentiated forms after 96 h of *in vitro* incubation with HIV PIs at 10 µM. This effect was also observed when cells were incubated with these HIV PIs at 1 µM, but in a less extension for ritonavir, lopinavir and nelfinavir (**Fig. 4**). In addition, a complementary analysis was performed by flow cytometry, where it was possible to observe a significant percentage (38%) of cells expressing the metacyclic trypomastigote-specific gp82 surface antigen in parasites treated with HIV PIs at 10 µM for 96 h when compared to the non-treated parasites. In a representative set of this result, the treatment of parasite cells with nelfinavir was shown (**Fig. 5A**). Although the trypomastigote forms observed *in vitro* after HIV PI treatment had a typical fully elongated nucleus with a round kinetoplast at the posterior region of the parasite, the flagellum was detached from the cellular body (**Fig. 5B**). The appearance of these aberrant trypomastigotes was demonstrated to be dose-dependent for ritonavir, lopinavir and nelfinavir, reaching 20–30% of total trypomastigotes after HIV PIs treatment at 10 µM for 96 h (**Fig. 5C**). The epimastigote-into-trypomastigote differentiation of Y, CL Brener and INPA 4167 strains was not observed when these cells were treated with the same concentrations of HIV PIs used for clone Dm28c (data not shown).

### Effects of HIV PIs on aspartic peptidase

The proteolytic activity present in the cellular extract from clone Dm28c epimastigotes was able to cleave the cathepsin D substrate in a typical time-dependent fashion (data not shown). The results showed that all the HIV PIs tested reduced the proteolysis of the substrate in a typical dose-dependent manner after a period of 2 h of reaction, as demonstrated in **Table 2**.

**Table 2:** Effect of HIV PIs on the aspartic-type peptidase activity of *T. cruzi* clone Dm28c epimastigotes.

Inhibitors	Residual activity (%)^a^		
	10 µM	50 µM	100 µM
Ritonavir	63.2±5.9	19.1±3.8	5.2±1.1
Lopinavir	70.6±5.8	25.3±4.1	6.8±2.2
Nelfinavir	71.9±4.1	21.9±3.7	6.1±2.7
Saquinavir	60.1±6.4	18.1±2.3	3.8±0.9
Indinavir	74.2±6.9	28.5±4.6	6.4±2.6
Amprenavir	74.8±3.8	24.7±5.2	4.9±3.1

a Residual activity is correlated to the activity detected in non-treated control extract, for which the hydrolysis of cathepsin D substrate is considered as being 100%.

### Effects of HIV PIs on the expression of peptidases other than the aspartic-type

**Figure 6:**
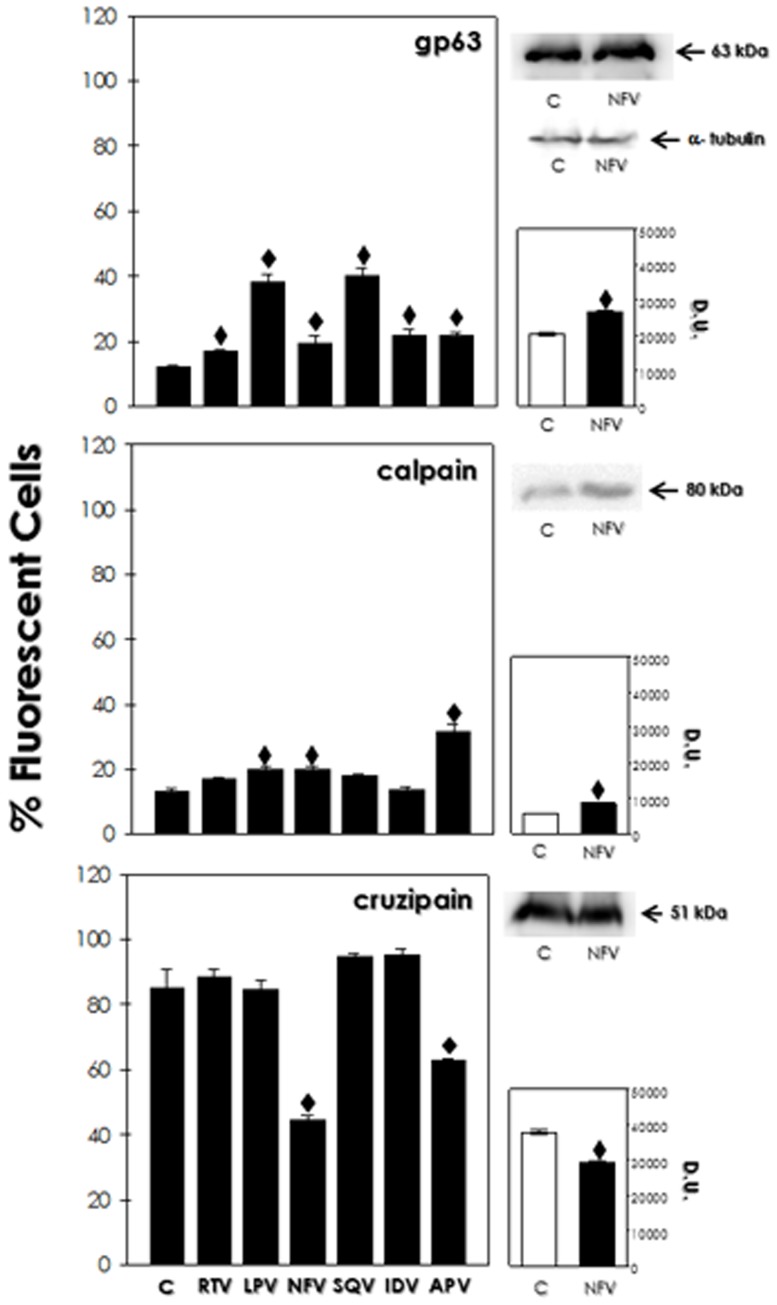
Effect of HIV PIs on the expression of gp63, calpain and cruzipain by *T. cruzi* clone Dm28c epimastigotes. At left, flow cytometric analysis of the anti-gp63, anti-Dm-calpain and anti-cruzipain antibodies binding to *T. cruzi* clone Dm28c epimastigotes after treatment with HIV PIs (IC_50_ value) for 24 h. **C**, non-treated cells; **RTV**, ritonavir; **LPV**, lopinavir; **NFV**, nelfinavir; **SQV**, saquinavir; **IDV**, indinavir; **APV**, amprenavir. Data are expressed as the percentage of fluorescent cells. At right, Western blotting showing the proteins recognized by the anti-gp63, anti-Dm-calpain and anti-cruzipain antibodies in the whole cellular extract from *T. cruzi* clone Dm28c epimastigotes in non-treated cells and after treatment with nelfinavir (IC_50_ value) for 24 h. Anti-α-tubulin monoclonal antibody was used as a control for sample loading in the blots. The apparent molecular masses of each band detected are shown, and the densitometric analysis of the reactive proteins is expressed as densitometric units (**D.U.**). The results represent means standard deviation of three independent experiments, and the symbol (♦) denotes statistic difference to control (*P*<0.05).

In this set of experiments, the expression of cruzipain, gp63 and calpain-like molecules was evaluated by flow cytometry in untreated and HIV PI-treated parasites. Generally, the metallopeptidase gp63 had its expression up-regulated significantly when parasites were subjected to the presence of HIV PIs (at IC_50_/72 h) for 24 h, especially lopinavir and saquinavir. Calpain-like molecules were found in higher levels in lopinavir-, nelfinavir- and mainly amprenavir-treated parasites. Contrarily, the major cysteine peptidase cruzipain was down-regulated when parasites were exposed to nelfinavir and amprenavir (**Fig. 6**). In order to corroborate the differential peptidase expression induced by HIV PIs on *T. cruzi*, Western blotting analysis was performed employing the same set of antibodies. To exemplify this set of experiments, we just presented the results obtained with the treatment of parasites with nelfinavir. In this context, a 63-kDa protein and an 80-kDa protein that displayed cross-reactivity with anti-gp63 and anti-Dm-calpain antibodies, respectively, had their expression significantly augmented after nelfinavir treatment, as judged by densitometric analysis. On the contrary, a significant decrease in the expression of the 55-kDa protein that cross-reacted with anti-cruzipain antibody was observed (**Fig. 6**).

### Effect of HIV PIs on the interaction with *R. prolixus*

**Figure 7:**
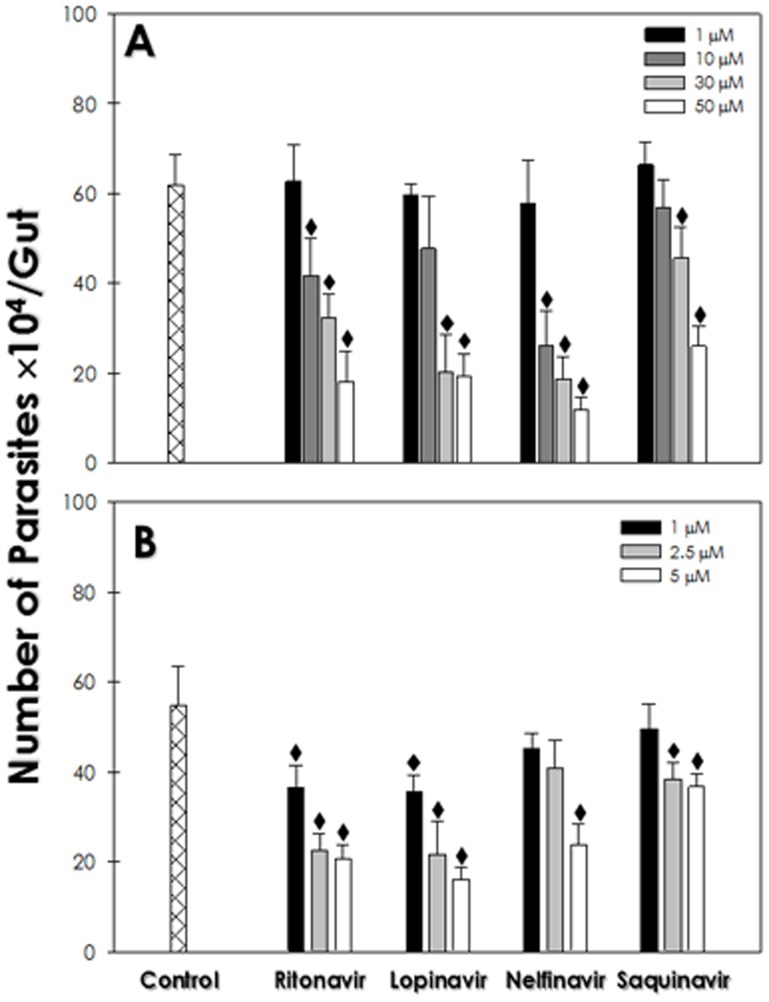
Effect of HIV PIs on the interaction of *T. cruzi* clone Dm28c epimastigote forms with *R. prolixus* explanted gut. Epimastigotes were pre-treated for 1 h (A) or 24 h (B) with different concentrations of HIV PIs and subjected to interaction with the gut of the insect vector. After 1 h of parasite-vector interaction, the intestines were washed and the attached forms were counted. Results are expressed as the number of adhered parasites per gut and the symbol (♦) denotes significant differences compared to control (*P*<0.05). All counts were performed in triplicate.

Initially, the parasites were treated for 1 h with ritonavir, lopinavir, nelfinavir or saquinavir, the four best HIV PIs able to block the *in vitro* proliferation, in concentrations ranging from 1 to 50 µM, and then the interaction with explanted guts from *R. prolixus* was performed. The results showed a dose-dependent inhibition of the adhesion process. At the highest concentration used (50 µM) for 1 h, all the HIV PIs used impaired the parasite-gut binding by approximately 81%, 71%, 69% and 58% for nelfinavir, ritonavir, lopinavir and saquinavir, respectively, in comparison to the control (**Fig. 7A**). When the parasites were treated with lower concentrations of the HIV PIs for a prolonged period of time (24 h), all the compounds used presented inhibition values statistically different from the control, with ritonavir, nelfinavir and lopinavir being more effective than saquinavir (**Fig. 7B**). The pre-treatment for 1 h with higher concentration of HIV PIs or for 24 h with lower dosages did not alter the viability of the parasites (data not shown).

### Effects of HIV PIs on trypomastigote viability

**Figure 8:**
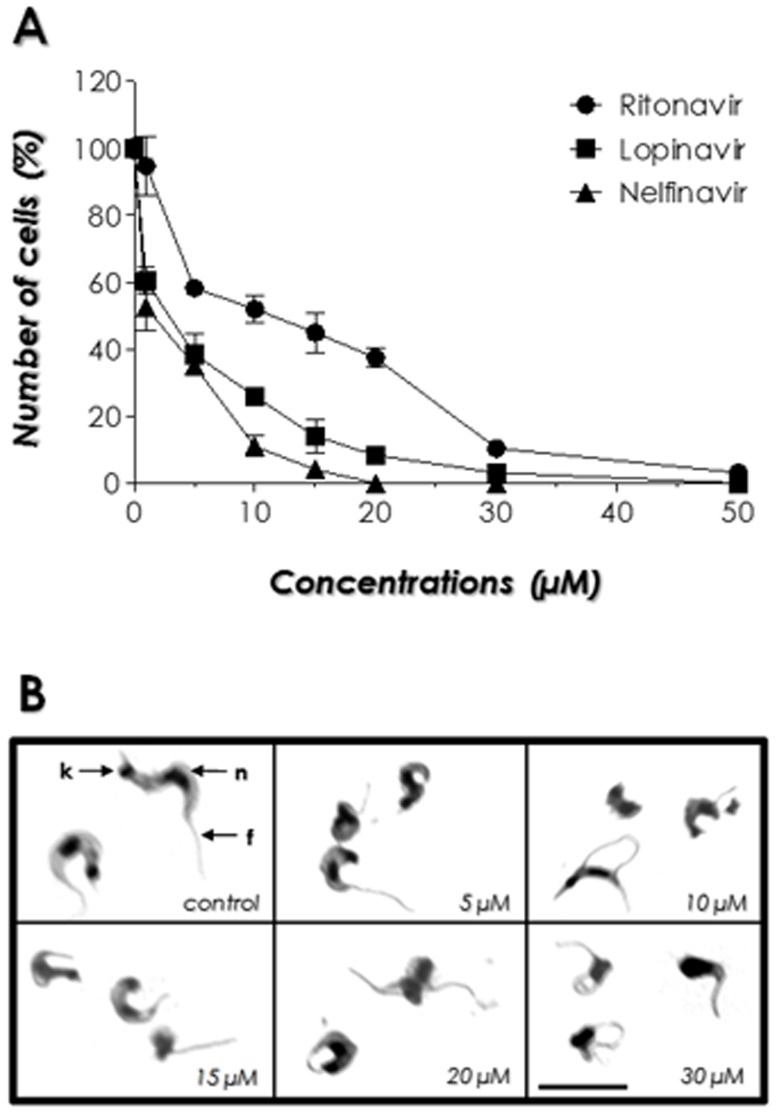
Effects of HIV PIs nelfinavir, lopinavir and ritonavir on *T. cruzi* clone Dm28c trypomastigote forms. (A) Viability rate of trypomastigotes after HIV PIs treatment. The assays were performed in the absence (control) and presence of HIV PIs at concentrations ranging from 1 to 50 µM for 24 h. Viable cells were counted by Trypan blue exclusion and motility in a Neubauer chamber. (B) Giemsa-stained smears of *T. cruzi* trypomastigote cells incubated in the absence (control) or in the presence of ritonavir at different concentrations for 24 h. Trypomastigote forms present a kinetoplast (**k**) in the posterior end of the parasite, a central nucleus (**n**), an elongated cell body and a flagellum (**f**) attached along the length of the cell body. The microscopy images are representative of the treatment of trypomastigotes with the three HIV PIs used in this experiment. Data shown are the mean standard error (S.E.) of three independent experiments performed in triplicate.

The HIV PIs nelfinavir, lopinavir and ritonavir were effective in reducing the viability of trypomastigotes in a dose-dependent manner after 24 h of treatment. Nelfinavir and lopinavir were the most effective HIV PIs tested, presenting LD_50_ values of 2.7 and 3 µM, respectively, while ritonavir reduced the trypomastigote viability with a LD_50_ value of 10.7 µM (**Fig. 8A**). DMSO, the solvent of HIV PIs, was also used as a control and did not affect the parasites viability (not shown). Light microscopy analysis of trypomastigotes treated with the HIV PIs at different concentrations revealed some morphological alterations in comparison to the typical control cells: parasites became round in shape, with reduced cell size, swollen of the cellular body and flagellum detachment. In a representative set of these results, the treatment of parasite cells with ritonavir was shown (**Fig. 8B**).

## Discussion

In the present work, we began to study the implications of different HIV PIs on the cell biology of *T. cruzi*, and simultaneously to explore the possible roles of aspartic peptidases in the life cycle of this parasite. HIV PIs are the only aspartic peptidase inhibitors licensed for use in humans, specifically in AIDS. Nowadays, AIDS treatment is performed with a HIV PI in combination with at least two other antiretroviral inhibitors in the so-called HAART (highly active antiretroviral therapy), and as a consequence, patients with HIV infection are living longer and with an improved quality of life [23]. The demonstration that HIV PIs have advantageous effects in some opportunistic infections caused by fungi and also some protozoa was associated not only with the restoration of the immune system of the host, but also due to a direct action on pathogens, as widely demonstrated in the literature [11], [24], [25].

In trypanosomatids, it has been observed that the aspartic peptidase inhibitors commonly used in HAART were capable of suppressing the proliferation of *Leishmania* species [26]-[28]. In an initial approach to study the effects of aspartic peptidase inhibitors against *T. cruzi*, we have previously demonstrated that the classic aspartic peptidase inhibitor pepstatin A was able to inhibit *T. cruzi* clone Dm28c epimastigote growth in a typical dose-dependent manner, with an IC_50_ value of 36.2 µM after 96 h [12]. In the present work, we showed that HIV PIs inhibited the proliferation of *T. cruzi* clone Dm28c epimastigotes at much lower concentrations than pepstatin A, being ritonavir, lopinavir and nelfinavir the most effective compounds tested. When the same HIV PIs were tested against distinct *T. cruzi* strains (Y, INPA 4167 and CL Brener), they inhibited the parasite proliferation, although significant differences were found in the IC_50_ values (**Table 1**). Our results are in accordance with the great variability within *T. cruzi* [15]. In this sense, it has been established that strains from distinct phylogenetic lineages present significant phenotypic differences among themselves, including the expression of peptidases, for example cruzipain [29]. The same type of variability has also been observed in treating several *T. cruzi* strains with nitro-derivative compounds [30], [31]. Different effects of distinct HIV PIs were also observed on the growth of several *Leishmania* species and/or strains [26]–[28].

The HIV PIs induced several alterations on epimastigote forms similar to those caused by pepstatin A, a prototypal aspartic peptidase inhibitor [12]. Due the morphological alterations observed after treatment with the HIV PIs by light microscopy, we aimed to analyze the *T. cruzi* clone Dm28c epimastigotes alterations more deeply by means of transmission electron microscopy. The treatment with the six HIV PIs revealed some peculiar changes in distinct cellular structures, such as flagellar membrane, mitochondrion and reservosomes, which could culminate in parasite death. Some of these ultrastructural changes are suggestive of different types of programmed cell death (PCD) that includes phenotypically distinct processes such as apoptosis, autophagy and necrosis [32], [33]. In this context, the major changes observed in all treatments were the damages in the cytoplasmic membrane, where the blebbing formation suggests apoptosis [34]. Some of the ultrastructural alterations, such as the formation of concentric membrane structures and profiles of endoplasmic reticulum are suggestive of autophagy. The mitochondrial swelling and vacuolization are common in apoptosis and necrosis, but the presence of membrane structures within the mitochondrion may indicate degradation by autophagy [34]. In *L. amazonensis*, ultrastructural alterations observed after treatment with nelfinavir and lopinavir, such as increase in the number of vesicles and wrapping of the nucleus by the endoplasmic reticulum, are also suggestive of autophagic event. On the other hand, lopinavir also induced a chromatin condensation, which is one of the features of apoptosis [35]. This drug is also effective in generating oxidative stress in *L. donovani*, leading to altered physiological parameters such as increase in the sub-G1 DNA content, nuclear DNA fragmentation and loss of mitochondrial potential, which are all characteristics of apoptosis [36]. We may speculate that depending on the intensity of induction (in this case, HIV PIs) the parasite can get into distinct types of PCD by cross-signaling, as already reported for mammalian cells [32], [37]. In fact, some data in the literature demonstrate the effect of diverse compounds against trypanosomatids that culminate in distinct death phenotypes. For instance, it was observed that lipid synthesis inhibitors, naphthoquinones, natural products and cytoskeletal inhibitors led to a pool of alterations that, in each case, are suggestive of different kinds of PCD [17], [34], [38]–[41]. However, mitochondrion is part of a convergent target of their mechanism of action, being an organelle commonly affected by these compounds [42].

An intriguing fact observed in the viability assays was a high rate of differentiation of *T. cruzi* clone Dm28c epimastigotes into morphological forms typical of trypomastigotes along the treatment with HIV PIs at 1 and 10 µM. Under the conditions performed in our experiments, these trypomastigote forms were visualized only in light microscopy, and not in transmission electronic microscopy, since the latter was performed after 72 h of treatment with HIV PIs at IC_50_ values. After 72–96 h of incubation, ritonavir, lopinavir, nelfinavir and saquinavir were the major inducers of the differentiation process. Confirming this finding, the gp82 molecule, which is a specific antigen of trypomastigote form, was detected in significant level in parasites after treatment with HIV PIs. However, many of these parasites presented morphological alterations, mainly the detachment of the flagellum from the cell body. The same differentiation process and the consequent alterations were seen in pepstatin A-treated parasites [12]. The trypomastigote flagellum is known to contribute to the cell movement and adhesiveness [18]. Bisaggio and co-workers [43] showed that suramin induced the flagellar detachment, and as a consequence its movement was not transmitted to the cell body, which reduced the parasite capability to interact with LLC-MK_2_ cells. *In vivo*, metacyclogenesis occurs in response to different stimuli, such as nutritional deficit and different molecules present in the triatomine intestine, such as feces and urine [44]. This signaling leads to a cascade of highly orchestrated cellular events involving a large number of molecules, among them numerous peptidases, that culminates in several molecular, physiological and morphological changes, leading to the emergence of a new cell shape [44]. A hypothesis to consider in our experiments may be the fact that HIV PIs could cause a stress that culminated in the parasite differentiation. *In vitro*, it has been demonstrated that the differentiation process of clone Dm28c epimastigotes into trypomastigotes, but not of other *T. cruzi* strains, is possible using chemically defined medium, which made this strain a valuable tool for the study of metacyclogenesis [45]. In fact, this differentiation process was previously demonstrated in *T. cruzi* epimastigotes under inhibitors pressure [12], [46]. Another aspect to consider would be the involvement of aspartic peptidases in metacyclogenesis, in which the inhibition of the proteolytic activity could trigger the process. In this sense, Alves and co-workers [47] demonstrated that the aspartic peptidase activity was decreased during the induction processing of promastigote into amastigote in *L. amazonensis*. However, cellular differentiation is not fully understood, as it involves several signaling pathways, which will require further investigation.

Based on our evidences, we also assessed whether the HIV PIs would induce any change in the peptidase expression by *T. cruzi,* since peptidases play an important role in many biological steps such as replication, nutrition, adhesion, infectivity and differentiation [9]. Interestingly, the treatment of *T. cruzi* epimastigotes with all the HIV PIs tested promoted an increased expression on surface gp63-like molecules, while lopinavir, nelfinavir and amprenavir promoted the increased detection of calpain-like molecules. A decrease in cruzipain levels was observed after treatment with amprenavir and mostly by nelfinavir. These changes were also observed after treatment with pepstatin A [12]. A possible explanation for the increased expression of gp63-like and calpain-like proteins could be that these inhibitors are directly affecting the aspartic peptidase activity, and as a physiological form of compensation, other parasite peptidases are expressed in greater quantity [48]. An alternative hypothesis could be that HIV PIs are exerting other non-specific effects on epimastigotes, leading to changes in the gene expression of the parasite in order to compensate the inhibition of the aspartic peptidase activity. A similar compensatory mechanism was reported in *L. amazonensis*, in which both gp63 and cysteine peptidase b (cpb) were up-regulated after treatment with nelfinavir, amprenavir and lopinavir [35]. In *T. cruzi*, the treatment of epimastigotes with MDL28170, a calpain inhibitor, induced an augmentation in the cruzipain levels [16]. This same inhibitor also reduced the metacyclogenesis of clone Dm28c, demonstrating the possible role of calpain-like molecules in this process [49], which was previously suggested by Giese and co-workers [50]. In addition, we may speculate that the lowest expression of cruzipain after nelfinavir treatment may be associated to the generation of the highest levels of altered trypomastigotes, since metacyclogenesis involves a considerable intracellular reorganization, in which cruzipain takes part [51].

Whereas ultrastructural alterations, an increased rate of differentiation and altered peptidases expression were detected after HIV PIs treatment upon *T. cruzi* epimastigotes, it became important to verify the consequences of these treatments on the interaction with the invertebrate host. After the blood meal and trypomastigote-into-epimastigote differentiation, *T. cruzi* epimastigotes migrate to the gut of the insect vector, where they divide and adhere strongly to perimicrovillar membranes in the posterior midgut [52]. The adhesion process of epimastigotes to these membranes is modulated by the participation of glycoconjugates exposed on the surface of the parasite, as glycoinositol phospholipids (GIPLs) and cruzipain [53], [54], but several proteins found in perimicrovillar membranes appear to be involved in this process as well [52]. However, the participation of other parasite surface molecules, such as gp72 [55], mucins [56] and Tcgp63 [57] cannot be excluded. The role of HIV PIs in this process was verified by checking the effect of pre-treatment of epimastigotes with the most effective HIV PIs on the interaction with *R. prolixus*. As we have noted, parasites pre-treated for 1 h with ritonavir, lopinavir, nelfinavir and saquinavir at higher doses were able to decrease the adhesion to the insect gut as efficiently as at lower concentrations of the same inhibitors but after 24-h incubation. In both situations, HIV PIs acted in a dose-dependent manner. One explanation for the decreased adhesion of epimastigotes pre-treated with HIV PIs to the *R. prolixus* intestinal epithelium could be the interference in the activity of aspartic peptidases, and as such these enzymes could participate in signaling, training and/or cleaving precursors of surface molecules. Indeed, our results demonstrated that amprenavir and nelfinavir induced a reduction on cruzipain-molecules. Alternatively, HIV PIs might affect the distribution and transport of surface molecules that will subsequently be anchored to the membrane and participate in the adhesion process. However, further investigations are needed to determine the actual role of HIV PIs on the interaction with the insect vector midgut.

It has been previously proposed that the effectiveness of HIV PIs in parasitic infections may be associated with their ability to modulate or block the cell proteasome [58] or their direct action on the aspartic peptidase activity produced by protozoa, as proposed in *Leishmania* spp. [27], [35], [47], [59], [60]. In this context, our work revealed that HIV PIs inhibited the hydrolysis of cathepsin D peptide substrate by cell extracts of *T. cruzi* clone Dm28c. Using the classic aspartic peptidase inhibitor pepstatin A, this inhibition was also observed over the same specific substrate, but in a more efficient way [12]. The genomic data of *T. cruzi* reported that two aspartic peptidases, which have homology with a signal peptide peptidase and presenilin, are classically inhibited by pepstatin A; genes for enzymes belonging to the pepsin family have not been found. However, the purified aspartic peptidases cruzipsin I and II were strongly blocked by inhibitors of pepsin family of peptidases, which includes peptatin A [9], [14]. While it may be reasonable to assume that the major target of HIV PIs in *T. cruzi* is the aspartic peptidases, the possibility of other targets should also be considered and may be investigated.

Up to now few data are available in the literature in order to establish the possible functions of aspartic peptidases in *Leishmania* spp. [11], and even less in *T. cruzi* and on the possibility of employing HIV PIs in Chagas' disease treatment. In this subject, although there are some studies concerning *T. cruzi*/HIV co-infection, some peculiarities about its epidemiology, pathogenesis, prophylaxis and especially its treatment remain unclear and undefined [3], [6]. The results presented in this work act collectively to help understanding the possible roles of aspartic peptidases and HIV PIs in *T. cruzi* biology and life cycle, and pave the way for the use of these proteolytic inhibitors as a possible alternative treatment for Chagas' disease, particularly in the chemotherapeutic handling of HIV-Chagas' disease co-infected patients. In this sense, our group tested three of these compounds – nelfinavir, lopinavir and ritonavir - on the clinically relevant trypomastigote forms of the disease. After only 24 h of treatment, our results showed that the LD_50_ values of HIV PIs for culture-derived trypomastigotes were significantly low. These PIs reduced drastically the parasite viability, causing many morphological alterations. Although the concentrations of HIV PIs reached in the blood plasma of patients under prolonged HAART treatment may vary by a great number of factors [61], these values are high enough to impair *T. cruzi* viability. These results reinforce the importance of the studies concerning the use of aspartic peptidase inhibitors against *T. cruzi.*
